# Therapeutic Reference Ranges for Psychotropic Drugs: A Protocol for Systematic Reviews

**DOI:** 10.3389/fpsyt.2021.787043

**Published:** 2021-11-24

**Authors:** Xenia M. Hart, Luzie Eichentopf, Xenija Lense, Thomas Riemer, Katja Wesner, Christoph Hiemke, Gerhard Gründer

**Affiliations:** ^1^Department of Molecular Neuroimaging, Medical Faculty Mannheim, Central Institute of Mental Health, Heidelberg University, Mannheim, Germany; ^2^Berlin Institute of Health, Institute of Clinical Pharmacology and Toxicology, Charité - Universitätsmedizin Berlin, Freie Universität Berlin, Humboldt-Universität zu Berlin, Berlin, Germany; ^3^Department of Psychiatry and Psychotherapy, Institute of Clinical Chemistry and Laboratory Medicine, University Medical Center of Mainz, Mainz, Germany

**Keywords:** psychotropic drugs, drug monitoring, therapeutic reference range, concentration/effect relationship, systematic review

## Abstract

**Background:** For many psychotropic drugs, monitoring of drug concentrations in the blood (Therapeutic Drug Monitoring; TDM) has been proven useful to individualize treatments and optimize drug effects. Clinicians hereby compare individual drug concentrations to population-based reference ranges for a titration of prescribed doses. Thus, established reference ranges are pre-requisite for TDM. For psychotropic drugs, guideline-based ranges are mostly expert recommendations derived from a conglomerate of cohort and cross-sectional studies. A systematic approach for identifying therapeutic reference ranges has not been published yet. This paper describes how to search, evaluate and grade the available literature and validate published therapeutic reference ranges for psychotropic drugs.

**Methods/Results:** Following PRISMA guidelines, relevant databases have to be systematically searched using search terms for the specific psychotropic drug, blood concentrations, drug monitoring, positron emission tomography (PET) and single photon emission computed tomography (SPECT). The search should be restricted to humans, and diagnoses should be pre-specified. Therapeutic references ranges will not only base upon studies that report blood concentrations in relation to clinical effects, but will also include implications from neuroimaging studies on target engagement. Furthermore, studies reporting concentrations in representative patient populations are used to support identified ranges. Each range will be assigned a level of underlying evidence according to a systematic grading system.

**Discussion:** Following this protocol allows a comprehensive overview of TDM literature that supports a certain reference range for a psychotropic drug. The assigned level of evidence reflects the validity of a reported range rather than experts' opinions.

## Introduction

Many psychotropic drugs have been in use for over 60 years. Great efforts have been made to individualize treatment with the available compounds (1). The only tool for such a personalization, which is now widely used in psychiatric clinical practice, is therapeutic drug monitoring (TDM). TDM-guided therapies aim at titrating drug levels in the blood within a range that is clinically helpful without causing harm. A key principle of TDM is the comparison of individual drug concentrations in the blood to a population-based reference range, the drug-specific therapeutic reference range. At concentrations below the lower limit of this range, a drug-induced response is unlikely to occur. Tolerability is expected to decrease at concentrations above the upper limit. Lower and upper limit of a reference range, respectively, should derive from well-designed clinical studies that relate measured drug concentrations to treatment response or specific adverse drug reactions. For many psychotropic drugs relationships between target engagement (TE) and drug blood concentrations on the one hand and clinical effects and side effects on the other hand are well-documented (2–4). TE by the respective drug (usually occupancy of neuroreceptors or transporters) can be quantified using molecular neuroimaging techniques like positron emission tomography (PET) and single photon emission computed tomography (SPECT). These studies supplement data from clinical studies in a meaningful manner. An overview of systematic reviews which aimed at finding therapeutic reference ranges, stated: “[W]e were not aware of a consensus on the optimum methodology for a systematic review that aims to determine upper and lower limits of the therapeutic range for a particular drug” (5). Inconsistent methodologies concerning the way that reference ranges were found have led to a high variation of ranges reported in the literature. In addition, current rating instruments are not designed to rate the quality of TDM studies. Understandably, this has led to criticism among clinicians, and reported ranges are more or less considered experts' opinions. As pointed out in a critical commentary, this holds also true for previously published TDM Consensus Guidelines that report therapeutic reference ranges for 154 neuropsychiatric drugs along with levels of recommendation for their clinical use (6–9).

## Materials and Methods

### Objective and Research Questions

This research protocol provides a tool for searching, evaluating and grading available literature in order to validate published therapeutic reference ranges for psychotropic drugs. Particular emphasis will be given to studies which investigate blood levels and clinical outcomes, such as response to drug treatment or adverse drug reactions. Studies on target engagement (usually receptor/transporter occupancy) from molecular neuroimaging can supplement the clinical evidence. The following research questions are addressed: Is there evidence for a concentration/response relationship and for a concentration/side effect relationship for a certain drug? Is there evidence that supports a lower or upper limit of a therapeutic reference range? How does the drug concentration relate to target engagement (usually receptor/transporter occupancy); and are these findings in line with the concentration/effect relationships and drug concentrations found in patients with psychiatric disorders receiving therapeutically effective doses? The authors may furthermore compute preliminary reference ranges from relevant studies, such as mean or median concentration ranges in patients with psychiatric disorders. This systematic review protocol follows the guidelines of the Preferred Reporting Items for Systematic Reviews and Meta-Analyses Protocols (PRISMA-P) (10) statement. Corresponding systematic reviews for four individual psychotropic drugs have been registered at the International Prospective Register of Systematic Reviews (PROSPERO; CRD42020215873, CRD42021216182, CRD42020218248, CRD42020215872).

### Search Strategy

The first step is a systematic search for relevant literature using established databases, such as MEDLINE, Web of Science, PsycINFO, and the Cochrane Library. Search terms for the relevant drug, blood concentrations, drug monitoring, PET and SPECT are helpful. No preset database search filters and no restrictions in regard to the publication date are to be applied. The search is complemented by a hand search in the reference lists of the included publications and in former published guidelines. An example of a search strategy for the antidepressant drug escitalopram is provided in the Supplementary Material.

### Eligibility Criteria

There are no restrictions in regard to the study design, e.g., both observational and interventional studies are included. Case reports and case series, however, are excluded. The search is restricted to humans, and relevant diagnoses have to be pre-specified, assuming that a specific reference range will only be valid for a particular indication. In order to be included in the evaluation of a certain concentration/effect relationship, studies must refer to patients with psychiatric disorders under monotherapy of the respective drug, meaning no other drug that mediates the relevant treatment effect should be administered concurrently. If at least one measurement was performed before the start of the new medication, the study will be considered for the computation of preliminary ranges only. Drug concentrations in blood should be measured after intake of the respective drug under steady-state conditions. Exceptions are made for molecular neuroimaging studies, which will be considered independent of the dosing period and diagnosis (studies with healthy volunteers included). Since studies investigating long-acting depot formulations are scarce, these studies will also be evaluated without regard to steady state conditions.

### Study Selection

After the removal of duplicates, screening of the literature has to be performed by two independent reviewers according to PRISMA guidelines. In cases where a final decision on the inclusion cannot be made based on the abstract alone, the full article must be reviewed. Any disagreements between the two reviewers must be resolved in a subsequent discussion. Inclusion and exclusion criteria are presented in Table 1. All studies that examine the drug blood concentrations in relation to clinical effect (without concomitant psychiatric medication), dose or target engagement have to be identified. Studies that did not ensure steady-state must be excluded (not necessarily applicable for imaging studies and studies with depot formulations). Studies performing population pharmacokinetic modeling analyses should be identified in the systematic review in order to discuss moderating factors on drug concentrations.

**Table 1:** Inclusion and exclusion criteria for study eligibility.

	**Inclusion criteria**	**Exclusion criteria**
Population	- Psychiatric patients treated with the respective psychotropic drug (not applicable for neuroimaging studies) - Main drug indications, which are specific to each drug, will be defined before the start of the review	- Non-human subjects, healthy volunteers, non-psychiatric patients - Post-mortem studies - Maternal use during pregnancy or lactation
Intervention	- Psychotropic monotherapy arm or period of observation (at least one blood level measurement before add-on therapy) - Treatment duration long enough to reach steady state (not applicable for neuroimaging studies and studies with depot formulations)	- Blood level is not measured in the steady state - Studies primarily comparing blood analysis techniques
Outcome(s)	- Drug concentrations measured in the blood (serum or plasma) - For concentration/effect studies: direct clinical outcome measures, i.e., safety or efficacy using a standardized rating scale (e.g., HAMD, MADRS, CGI)* - For neuroimaging studies: target engagement, usually by receptor or transporter occupancy	- No mean or median blood level reported
Study Design	- Observational and interventional studies are included - Reviews and meta-analyses investigating a concentration/ effect relationship for the relevant drug	- Reviews and experts' opinions - Gray literature - Case reports and case series
Other		- Papers containing the same data - No abstract available - Data from simulation studies

*Biomarkers (e.g., QTc-time) are not regarded a direct clinical outcome measure.

### Data Extraction

Both reviewers have to independently extract the following information from each study: lead author, year, title, country, study design, number and details of subjects, diagnosis, mean dose ± standard deviation (SD), mean blood concentration ± SD, concentration range, clinical efficacy or side effect measures, and main outcomes. Any disagreements between the reviewers have to be resolved in a subsequent discussion. Finally, if necessary, the authors of the original papers will also be contacted if further data is necessary for their interpretation.

### Quality Assessment

Reviewers have to independently (i) rate internal quality of included studies dependent of the study design (ii) assess the quality and reporting of TDM components of the studies. To date, there are no standardized quality tools for studies specifically investigating TDM or concentration/effect relationships. Therefore, we adjusted the quality criteria in a recent review by Kloosterboer et al. on the concentration/effect relationship of psychotropic drugs in minors (11), which were modified from a previously published meta-analysis by Ulrich et al. for haloperidol (12). A detailed description of the individual items can be found in the Supplementary Material. If a study does not completely report or implement an item, that item is rated insufficient. The TDM quality score ranges from 0 to 10 [selection (scale 0–3), comparability (scale 0–2), and drug monitoring (scale 0–5)]. For the quality assessment of cohort studies and cross-sectional studies, an adapted version of the Newcastle–Ottawa Scale (13) is used. The quality score ranges from 0 to 10 [selection (scale 0–4), comparability (scale 0–2), and outcome (scale 0–4)] for cohort studies and from 0 to 8 [selection (scale 0–4), comparability (scale 0–2), and outcome (scale 0–2)] for cross-sectional studies. Likewise, reviewers rate the quality of the relevant efficacy cohort of randomized controlled clinical trials separately using the Cochrane risk-of-bias tool for randomized trials (14). Any disagreements are resolved through discussion. Authors of the original papers will be contacted if further information is required.

### Considerations for the Quality Assessment of TDM Studies

#### Representativeness of the Patient Sample

For the study results to be applied in a generalized manner, it is important to have a representative sample, which reflects the target population of the resulting reference range. A study population only comprising of treatment-resistant patients or patients with side effects to another treatment does not reflect the general patient population and a resulting range is not transferable to “normal” patients. Likewise, a study population drawn from patients for whom genotyping has been demanded by the clinician will not reflect the target population. Patients 18 years and younger or 65 years and older should be compared with the average adult population. For some psychotropic drugs, ethnic variation in distribution in CYP expression patterns is relevant for the metabolism of the administered drug. This is especially important, if the main metabolite of the drug contributes to the pharmacologic action. A variation in the metabolite-to-parent compound ratio and thus, the sum of active and parent compound, may possibly influence clinical effects in these drugs. Since the evidence on this phenomenon is still very small, its clinical relevance should be revised for every substance individually. If an influence has been shown, studies must be evaluated in regard to the factor ethnicity. This holds also true for studies using variations in drug formulations or chemical forms (prodrugs). References ranges may not easily be transferred from originator products.

#### Diagnosis

To ensure comparability between studies, patients should be selected patients should be selected according to psychiatric and associated classification systems [of which the latest versions are the 5th edition of the American Psychiatric Association's (15) and the 11th edition of the World Health Organization's (16), which comes into effect in 2022]. Ideally, a homogeneous sample of patients according to one main diagnosis should be investigated. With a heterogeneous sample, a sub-analysis per relevant category should be provided. Differences in reference ranges across, usually related but also across unrelated, diagnosis should be emphasized in the final review.

#### Comedication

To avoid clinical effect bias, no drugs that potentially affect the treatment outcome should have been taken concomitantly during the study period. If detailed information on comedication was not provided, the study is rated as insufficient. The use of on-demand medication such as benzodiazepines or sleep medication must be considered adequate. Pre-medication should be registered as study characteristic and not be scored. For reviews about reference ranges of substances in which the active metabolite contributes to clinical efficacy and an altered metabolite to parent compound ratio might lead to a change in clinical efficacy, studies allowing concomitant drugs that interfere with the metabolism of the target drug should be identified.

#### Dose Design

The clinical status of a subject determines the amount of dose administered and thus the drug concentration. To avoid a possible reversal of a causal relationship resulting from such an effect, a study design with a fixed dose should be preferred over a design with a flexible dose (17). Flexible dosing is usually insufficient, since it may give rise to artificially negative correlations between concentrations and clinical effects (10).

#### Analytical Method for the Assay of Drug Concentration in Serum or Plasma

An analytical method is considered valid if it accurately, precisely, selectively, sensitively, reproducibly, and stably measures the concentration of the substance (9). In general, chromatographic methods, such as high-performance liquid chromatography (HPLC) and liquid chromatography-mass spectrometry (LC-MS), are selective and sensitive measurement methods. Immunoassays are considered low specific. The lower detection limit of the chosen analytical method should allow drug concentration measurements below the lower limit of currently recommended therapeutic reference ranges. Double measurements of samples are preferred, but they are not performed in clinical routine practice.

#### Blood Sample Collection

The time of sample collection affects the blood concentration of the drug. Sampling should be performed at steady-state, preferably at trough level since TDM-guided pharmacotherapy usually relies on minimal drug concentration, if not indicated otherwise. In clinical routine, blood withdrawal in the morning, before the first dose has been recommended (12–16 or 24 h after last dose) (9). Inconsistent sampling time points introduce bias; however considerably less likely for substances with long half-lives than for those with short elimination half-lives. Drug concentration of substances with long elimination half-lives (e.g., fluoxetine and aripiprazole), extended-release and depot formulations remain relatively stable over the day (18) and allow sampling within 12–24 h after the last drug intake. Sampling times should be described in publications when reporting drug concentrations. It is generally assumed that the steady-state condition is reached after 5 times the half-life of a drug. Drug sampling before the steady-state is reached, however, may result in an underestimation of clinical efficacy. This also holds true for long-acting depot medication.

#### Concentration Design

Correlations of measured serum concentrations with early response (e.g., after 1 week) is problematic, because of the well-described time lag between treatment initiation and onset of antidepressant/antipsychotic effects. The sampling schedule should include repeated sampling (at least two samples) in a patient over several weeks, ideally at different doses. In order to reflect a representative distribution of drug concentrations, a study's dose regimen should result in a sufficiently wide drug concentration range, with data of sub- and/or supratherapeutic drug concentrations.

## Results

### Reporting of Results

Results must be reported using the Preferred Reporting Items for Systematic Reviews and Meta-Analyses (PRISMA) guideline. The characteristics of all included studies (author/s, year, country, study design, intervention details, and study population details) must be displayed in a tabular summary.

### Grading of Evidence

The strength of available evidence for that supports a concentration/response relationship or concentration/ side effect relationship for a drug will be reflected by the assignment of a certain level. Grading into levels of evidence will be performed following the recommendations of the WFSBP guidelines for clinical guideline development (19). (i) Prioritize and evaluate (risk-of-bias assessment) single RCTs: when sufficient RCTs exist that support a certain concentration/effect relationship and these are of high quality and do not contradict each other, this approach is preferred. (ii) Evaluate meta-analyses (risk-of-bias assessment): when there are at least three RCTs for one treatment and these are inconsistent—meaning that some studies show a difference to placebo and others do not—meta-analyses of high quality should be used. (iii) Evaluate systematic reviews without meta-analysis (risk-of-bias assessment). This source of evidence should only be used if no recommendations can be generated from (1) and (2). It is not recommended to base the evidence grading on non-systematic reviews. Levels of evidence relating to the published literature are documented in Table 2. If evidence is found to support the relationship between drug concentration and therapeutic response (level A, strong or level B, limited), a valid therapeutic reference range, at least the lower limit, is likely to be found by an evaluation of the available data. The overall quality of evidence is reported as “strong,” “limited,” “low,” or “no evidence.”

**Table 2:** Grading into levels of evidence for a concentration/effect relationship following the recommendations of the WFSBP guidelines for clinical guideline development.

**Levels of evidence for concentration/effect relationship**		
**Evidence for a certain effect is:**	**Grade**	**Explanation**
**Strong**	A	At least two independent randomized clinical trials with a low risk of bias show a concentration/effect relationshipANDNo negative randomized clinical trials with a low risk of bias exist.If there are contradicting results from randomized clinical trials, the majority of randomized clinical trials AND/OR a meta-analysis with low risk of bias shows a relationship.
**Limited**	B	One randomized clinical trials with a moderate risk of bias showing a concentration/effect relationshipANDNo negative studies existORMeta-analyses with a moderate risk of bias that show a relationship.
**Low**	C	One or more prospective open studies (with a minimum of 10 evaluable patients per group) using a control group, but no randomization, or using no control group, show concentration/effect relationships.OROne or more well-conducted case control or cohort studies (with a minimum of 10 evaluable patients) with a moderate probability that the concentration/effect relationship is causal.ORRandomized clinical trials AND/OR meta-analyses with a high risk of bias show concentration/effect relationships.
**No evidence**	D	Insufficient data do not allow evaluation if a concentration/effect relationship existsOREvidence is given that a concentration/effect relationship does not exist (e.g., tranylcypromine, agomelatine)

### Data Synthesis

Concentration data must be pooled in order to find mean concentration ranges across studies. The theoretically expected concentration range in a patient population is estimated using data from a reference sample of patients, preferentially without co-medication or pharmacogenetic abnormalities. The pooled concentration, daily dose and C/D have to be combined and calculated using random-effect and fixed-effect models based on the *I*^2^ statistic. The *I*^2^ statistic has to be used to examine to presence of substantial heterogeneity between studies, with *I*^2^-values > 50% indicating heterogeneity. Subgroup analyses might be appropriate to examine the impact of moderating factors on concentration, such as patient populations with differing CYP expression patterns, age, sex or concomitant medications. In the next step, ranges of blood concentrations from only responders to a drug are computed to obtain a preliminary responder reference range for the psychotropic drug. There is no consistent method for calculating these ranges. We propose the use of mean ± one standard deviation (SD) or interquartile ranges (25th−75th percentiles) of drug concentrations in the blood.

## Discussion

Our strategy, on how to search and grade TDM-related literature, aims at finding therapeutic reference ranges for psychotropic drugs that are objectively evaluated. Each drug has to be assigned to a level according to the strength of evidence which refers to the underlying concentration/effect relationship. Methodology that has been used to uncover clinical response of psychotropic drugs in relation to blood concentration, however, is highly prone to failure (20). Concentration/response relationships are not well-established for most psychotropic drugs. As a consequence, many published ranges must be regarded as preliminary. In addition, published studies strongly differ in design and quality; their critical evaluation, as described here, is mandatory. This protocol introduces a standard on how to identify and grade evidence underlying therapeutic reference ranges. The methodology may be extended to other drug classes, since the lack of evaluated therapeutic reference ranges is not restricted to TDM in psychiatry.

## Data Availability Statement

The original contributions presented in the study are included in the article, further inquiries can be directed to the corresponding author.

## Author Contributions

XH developed the first draft of the protocol. CH and GG supervised the entire manuscript writing and contributed to the revision of the protocol. XL, KW, LE, and TR have contributed to the development of the search strategy and quality assessment criteria. All authors have read and approved the final manuscript.

## Conflict of Interest

CH has received speaker's fees from Otsuka. He is editor of PSIAC, a web-based platform analyzing pharmacokinetic and –dynamic drug interactions. The software is distributed by Springer Nature, Heidelberg, Germany. GG has served as a consultant for Allergan, Boehringer Ingelheim, Institute for Quality and Efficiency in Health Care (IQWiG), Janssen-Cilag, Lundbeck, Otsuka, Recordati, ROVI, Sage, and Takeda. He has served on the speakers' bureau of Gedeon Richter, Janssen Cilag, Lundbeck, Otsuka, Recordati. He has received grant support from Boehringer Ingelheim, Lundbeck and Saladax. He is co-founder and/or shareholder of Mind and Brain Institute GmbH, Brainfoods GmbH, OVID Health Systems GmbH and MIND Foundation gGmbH. The remaining authors declare that the research was conducted in the absence of any commercial or financial relationships that could be construed as a potential conflict of interest.

## Publisher's Note

All claims expressed in this article are solely those of the authors and do not necessarily represent those of their affiliated organizations, or those of the publisher, the editors and the reviewers. Any product that may be evaluated in this article, or claim that may be made by its manufacturer, is not guaranteed or endorsed by the publisher.
