# Supplementary material for: Therapeutic Reference Ranges for Psychotropic Drugs: A Protocol for Systematic Reviews
Source: Front Psychiatry. 2021 Nov 24;12:787043. doi: 10.3389/fpsyt.2021.787043 (PMC8653700; doi:10.3389/fpsyt.2021.787043)
Supplement: Supplementary file 1 [file Data_Sheet_1.docx]

Supplementary Material “Therapeutic Reference Ranges for Psychotropic Drugs: a Protocol for Systematic Reviews”

[Example search strategy for the antidepressant drug escitalopram 2](#_Toc83909918)

[S1 General quality criteria for the therapeutic drug monitoring component (Total 10 points) 3](#_Toc83909919)

[S2 Study type specific quality assessment modified from the Newcastle-Ottawa Quality Assessment Scale - Cohort studies (Total 10 points) 5](#_Toc83909920)

[S3 Study type specific quality assessment modified from the Newcastle-Ottawa Quality Assessment Scale - Cross sectional studies (Total 8 points) 7](#_Toc83909921)

# Example search strategy for the antidepressant drug escitalopram

| PubMed |
| --- |
| ("Escitalopram"[tw] OR "S Citalopram"[tw] OR "Cipralex"[tw] OR "Lexapro"[tw] OR "Seroplex"[tw]) AND ("serum level*"[tw] OR "plasma level*"[tw] OR "blood level*"[tw] OR "drug level*"[tw] OR "serum concentration*"[tw] OR "plasma concentration*"[tw] OR "blood concentration*"[tw] OR "drug concentration*"[tw] OR "Drug Monitoring"[Mesh] OR "drug monitor*"[tw] OR “positron emission tomography”[MeSH Terms] OR "Positron Emission Tomogra*"[tw] OR "PET scan*"[tw] OR "Tomography, Emission Computed, Single Photon"[Mesh] OR "Single Photon Emission*"[tw] OR "SPECT"[tw] OR "CAT Scan"[tw] OR "single photon emission computed tomography computed tomography"[MeSH Terms]) NOT ("Animals"[MeSH Terms] NOT "humans"[MeSH Terms]) |
| Web of Science |
| (TS=Escitalopram OR TS="S Citalopram" OR TS=Cipralex OR TS=Lexapro OR TS=Seroplex) AND (TS =(serum NEAR/1 level*) OR TS = (plasma NEAR/1 level*) OR TS= (blood NEAR/1 level*) OR TS=(drug NEAR/1 level*) OR TS=(serum NEAR/1 concentration*) OR TS=(plasma NEAR/1 concentration*) OR TS=(blood NEAR/1 concentration*) OR TS=(drug NEAR/1 concentration*) OR TS=(drug NEAR/1 monitor*) OR TS=(positron NEAR/1 emission NEAR/1 tomogra*) OR TS=(PET NEAR/1 scan*) OR TS=(single NEAR/1 photon NEAR/1 emission*) OR TS=SPECT OR TS=(CAT NEAR/1 Scan)) |
| Cochrane Library |
| ("Escitalopram" OR "S Citalopram" OR "Cipralex" OR "Lexapro" OR "Seroplex") AND ([mh "positron emission tomography"] OR [mh "Tomography, Emission-Computed, Single-Photon"] OR [mh "single photon emission computed tomography computed tomography"] OR (positron NEAR/1 emission NEAR/1 tomogra* ) OR (PET NEAR/1 scan*) OR (tomography, emission NEAR/1 computed, single NEAR/1 photon) OR (single NEAR/1 photon NEAR/1 emission*) OR SPECT OR (CAT NEAR/1 Scan) OR (single NEAR/1 photon NEAR/1 emission) OR (single NEAR/1 photon NEAR/1 emission NEAR/1 computed NEAR/1 tomography NEAR/1 computed NEAR/1 tomograph*):ti,ab,kw OR (drug NEAR/1 monitor*):ti,ab,kw OR (serum NEAR/1 level*) OR (plasma NEAR/1 level*) OR (blood NEAR/1 level*) OR (drug NEAR/1 level*) OR (serum NEAR/1 concentration*) OR (plasma NEAR/1 concentration*) OR (blood NEAR/1 concentration*) OR (drug NEAR/1 concentration*)):ti,ab,kw |
| PsycINFO |
| ("Escitalopram" OR "S Citalopram" OR "Cipralex" OR "Lexapro" OR "Seroplex") AND (MA "positron emission tomography" OR "positron emission tomogra*" OR "pet scan*" OR MA "tomography, emission computed, single photon" OR "single photon emission*" OR "SPECT" OR "CAT Scan" OR MA "single photon emission computed tomography computed tomography" OR MA "Drug Monitoring" OR "Drug Monitoring" OR "serum level*" OR "plasma level*" OR "blood level*" OR “drug level*" OR "serum concentration*" OR "plasma concentration*" OR "blood concentration*" OR “drug concentration*") NOT (MA "Animals" NOT MA "humans") |

**S1 General quality criteria for the therapeutic drug monitoring component (Total 10 points)**

| **Selection (Maximum 3 points):**   1. Representativeness of the patient sample (Maximum 1 point): 2. Truly representative of the average patient population in the community (referred to as average Caucasian patient population) * 3. Somewhat representative of the average patient population in the community * 4. Selected group of users or user not representative for review outcome 5. No description of the derivation of the cohort   For b) “Somewhat representative”: i) A study population only comprises of patients for whom TDM was requested by the clinician. ii) Patients are drawn from an ethic group with a different distribution in CYP expression patterns than Caucasians, which are relevant for the metabolism of the administered drug and the main metabolite does not contribute to the pharmacologic action.  For c) “Selected group of users”: i) Patients are drawn from an ethic group with a different distribution in CYP expression patterns than Caucasians, which are relevant for the metabolism of the administered drug and the main metabolite contributes to the pharmacologic action. ii) A study population only comprises of treatment-resistant patients or patients with side effects to another treatment iii) A study population only comprises of patients for whom genotyping has been demanded by the clinician. iii) A study population only comprises of patients 65 years and older or 18 years and younger.   1. Diagnosis (Maximum 2 points): 2. Patients selected according to psychiatric classifications and associated classification system are reported * 3. Homogenous sample according to one main diagnosis, healthy controls or: With a heterogeneous sample, a sub analysis per relevant category should be provided * 4. no description of the patient classification or heterogeneous sample in regard to diagnosis   **Comparability (Maximum 2 points):**  For reviews about reference ranges in those active metabolite contributes to clinical efficacy and an altered metabolite to parent compound ratio might lead to a change in clinical efficacy:   1. Comedication (Maximum 1 point): 2. If clinical effects are assessed: No drug that influences the investigated clinical effect (e.g. antidepressant or antipsychotic effect) or metabolism of the drug (clinically relevant) under study is taken simultaneously, or: A sub analysis/correction is provided (Medication on demand, e.g. Benzodiazepines or sleep medication, is permitted) * 3. If no clinical effects are assessed: No drug that influences the metabolism of the drug (clinically relevant) under study is taken simultaneously, or: A sub analysis/correction is provided * 4. No or insufficient information that allows to assess possible influence of administered comedication is given   For reviews about reference ranges in those only the parent compound contributes to clinical efficacy:   1. Comedication (Maximum 1 point): 2. If clinical effects are assessed: No drug that influences the investigated clinical effect (e.g. antidepressant or antipsychotic effect) of the drug under study is taken simultaneously, or: A sub analysis/correction is provided * 3. If no clinical effects are not assessed: information on administered comedication is given * 4. No or insufficient information that assess possible influence of administered comedication is given 5. Dose design (Maximum 1 point): 6. Fixed doses were administered * 7. Flexible dosing was performed 8. Single doses were administered or no information on dosing strategy   Flexible dosing describes the adaption of doses according to the clinician’s decision, in case of side effects or insufficient tolerability.  **Drug Monitoring (Maximum 5 points):**   1. Analytical method for the assay of drug concentration in serum or plasma (Maximum 1 point) 2. Validated analytical method with appropriate limit of quantification * 3. Not validated analytical method was used 4. Insufficient description or no validated analytical method used |
| --- |
| 1. Blood sample collection (Maximum 2 points) 2. Plasma or serum concentrations are in the steady state * 3. Time interval between sampling and drug intake described or sampling at trough * 4. Insufficient description 5. Steady-state not reached |
| 1. Concentration design (Maximum 2 points) 2. A schedule with frequent measurements (at least 2) of blood samples was used * 3. Sufficiently broad concentration range including sub- and/or supratherapeutic drug concentrations (in the steady state) according to former recommended reference ranges * 4. Single concentration measurements 5. No sufficiently broad concentration range   This scale has been adapted from a published systematic review by Kloosterboer^1^ et al. to perform a comparable quality assessment across study types for the systematic review. A comparable rating across studies is needed in order to decide, which studies can be included in the data synthesis. A standardized rating scale has not been established in the literature yet.  **S2 Study type specific quality assessment modified from the Newcastle-Ottawa Quality Assessment Scale - Cohort studies (Total 10 points)** |

| **Selection (Maximum 4 points):**   1. Representativeness of the Exposed Cohort (Maximum 1 point):   a) Truly representative of the average patient population in the (referred to as average Caucasian patient population) *  b) Somewhat representative of the average patient population in the community *  c) Selected group of users or user not representative for review outcome  d) No description of the derivation of the cohort  Item is assessing the representativeness of exposed individuals in the community, not the representativeness of the sample of women from some general population. For example, subjects derived from groups likely to contain middle class, better educated, health oriented women are likely to be representative of postmenopausal estrogen users while they are not representative of all women (e.g. members of a health maintenance organisation (HMO) will be a representative sample of estrogen users. While the HMO may have an under-representation of ethnic groups, the poor, and poorly educated, these excluded groups are not the predominant users of estrogen).  “Somewhat representative”: i) A study population only comprises of patients for whom TDM was requested by the clinician. ii) Patients are drawn from an ethic group with a different distribution in CYP expression patterns than Caucasians, which are relevant for the metabolism of the administered drug and the metabolite does not contribute to the pharmacologic action.  “Selected group of users”: i) Patients are drawn from an ethic group with a different distribution in CYP expression patterns than Caucasians, which are relevant for the metabolism of the administered drug and the metabolite does contribute to the pharmacologic action. ii) A study population only comprises of treatment-resistant patients or patients with side effects to another treatment iii) A study population only comprises of patients for whom genotyping has been demanded by the clinician. iii) A study population only comprises of patients 65 years and older or 18 years and younger.   1. Selection of the Control Cohort (Maximum 1 point): 2. Drawn from the same community as the exposed cohort * 3. Drawn from a different source 4. No description of the derivation of the non exposed cohort 5. No control cohort 6. Ascertainment of Exposure (Drug Intake) (Maximum 1 point): 7. Secure record (e.g. Adherence problems detected by blood level measurement or pill counting and discussed by the authors) * 8. Study record (e.g. drug intake documented by study personal) * 9. Patient self-report (e.g. patient diary) 10. No description or no record 11. Demonstration That Outcome of Interest Was Not Present at Start of Study (Maximum 1 point): 12. Yes (Outcome of most interest according to the authors) * 13. No 14. Not applicable |
| --- |
| **Comparability (Maximum 2 points):**   1. Comparability of “exposed” and “non-exposed” individuals or of outcome groups 2. The study controls for the most important factor * 3. The study controls for any additional factor *   Either exposed and non-exposed individuals must be matched in the design and/or confounders must be adjusted for in the analysis. Statements of no differences between groups or that differences were not statistically significant are not sufficient for establishing comparability. Note: If the relative risk for the exposure of interest is adjusted for the confounders listed, then the groups will be considered to be comparable on each variable used in the adjustment. There may be multiple ratings for this item for different categories of exposure (e.g. ever vs. never, current vs. previous or never). (Examples for factors controlled by study design: comedication, premedication and washout-phase. Examples for factors controlled by analysis: Mean doses if flexible design, sex, age and baseline severity of illness). |
| **Outcome (Maximum 4 points):**   1. Assessment of outcome (Maximum 1 point): 2. Independent or blind assessment stated in the article, or confirmation of the outcome by reference to secure records (e.g. receptor occupancy by positron emission tomography) * 3. Record linkage * 4. Self-report (i.e. self-rating scales or non-established rating scales) 5. No description or insufficient information   For some outcomes (e.g. genotypes, blood concentrations), reference to the medical record is sufficient to satisfy the requirement for confirmation. This would not be adequate for clinical efficacy outcomes where a structured rating scale would be required. For neuroimaging studies, which also assess clinical effects, both methods will be evaluated and the lowest rating will be used.   1. Was Follow Up Long Enough for Outcomes to Occur (Maximum 1 point) : 2. yes (select an adequate follow up period for outcome of interest ) * 3. no 4. Not applicable 5. Adequacy of Follow Up of Cohorts (Maximum 1 point) 6. Complete follow up - all subjects accounted for * 7. Subjects lost to follow up unlikely to introduce bias - small number lost - ≥ 5 % follow up, or description provided of those lost indicates no bias (see Cochrane Tool RoB 2.0 Item 3.2, 3.3 and 3.4)) * 8. Follow up rate < 95% and no description of those lost 9. no statement 10. Statistical test (Maximum 1 point): 11. The statistical test used to analyze the data is clearly described and appropriate, and the measurement of the association is presented, including confidence intervals and the probability level (p value) * 12. The statistical test is not appropriate, not described or incomplete |

| This scale has been adapted from the Newcastle-Ottawa Quality Assessment Scale^2^ for cohort studies. We have not selected one factor that is the most important for comparability, because the variables are not the same in each study. Thus, the principal factor should be identified for each study. The resulting quality score can be used to compare risk of bias across cohort studies in our review.  **S3 Study type specific quality assessment modified from the Newcastle-Ottawa Quality Assessment Scale - Cross sectional studies (Total 8 points)** |
| --- |
| **Selection (Maximum 4 points):**   1. Representativeness of the sample (Maximum 1 point):   a) Truly representative of the average in the target population (referred to as average Caucasian patient population) *  b) Somewhat representative of the average in the target population *  c) Selected group of users or user not representative for review outcome  d) No description of the sampling strategy  Item is assessing the representativeness of exposed individuals in the community, not the representativeness of the sample of women from some general population. For example, subjects derived from groups likely to contain middle class, better educated, health oriented women are likely to be representative of postmenopausal estrogen users while they are not representative of all women (e.g. members of a health maintenance organisation (HMO) will be a representative sample of estrogen users. While the HMO may have an under-representation of ethnic groups, the poor, and poorly educated, these excluded groups are not the predominant users of estrogen).  “Somewhat representative”: i) A study population only comprises of patients for whom TDM was requested by the clinician. ii) Patients are drawn from an ethic group with a different distribution in CYP expression patterns than Caucasians, which are relevant for the metabolism of the administered drug and the metabolite does not contribute to the pharmacologic action.  “Selected group of users”: i) Patients are drawn from an ethic group with a different distribution in CYP expression patterns than Caucasians, which are relevant for the metabolism of the administered drug and the metabolite does contribute to the pharmacologic action. ii) A study population only comprises of treatment-resistant patients or patients with side effects to another treatment iii) A study population only comprises of patients for whom genotyping has been demanded by the clinician. iii) A study population only comprises of patients 65 years and older or 18 years and younger.   1. Sample size (Maximum 1 point): 2. A priori sample size calculation justified and satisfactory * 3. Sample size not justified or not satisfactory 4. Non-respondents (Maximum 1 point): 5. Comparability between respondents and non-respondents characteristics is established, and the response rate is satisfactory (e.g. responders/ nonresponders, genotype groups, comedication groups) * 6. The response rate is unsatisfactory, or the comparability between respondents and non-respondents is unsatisfactory 7. No description of the response rate or the characteristics of the responders and the non-responders 8. Ascertainment of Exposure (Drug Intake) (Maximum 1 point): 9. Secure record (e.g. Adherence problems detected by blood level measurement or pill counting and discussed by the authors) * 10. Study record (e.g. drug intake documented by study personal) * 11. Patient self-report (e.g. patient diary) 12. No description or no record   **Comparability (Maximum 2 points):**   1. Comparability of outcome groups (Maximum 2 points): 2. The study controls for the most important factor * 3. The study control for any additional factor *   The subjects in different outcome groups are comparable, based on the study design or analysis. Confounding factors are controlled. (Examples for factors controlled by study design: comedication, premedication and washout-phase. Examples for factors controlled by analysis: Mean doses if flexible design, sex, age and baseline severity of illness).  **Outcome (Maximum 2 points):**   1. Assessment of outcome (Maximum 1 point): 2. Independent or blind assessment stated in the article, or confirmation of the outcome by reference to secure records (e.g. receptor occupancy by positron emission tomography) * 3. Record linkage * 4. Self-report (i.e. self-rating scales or non-established rating scales) 5. No description or insufficient information   For some outcomes (e.g. genotypes, blood concentrations), reference to the medical record is sufficient to satisfy the requirement for confirmation. This would not be adequate for clinical efficacy outcomes where a structured rating scale would be required. For neuroimaging studies, which also assess clinical effects, both methods will be evaluated and the lowest rating will be used.   1. Statistical test (Maximum 1 point): 2. The statistical test used to analyze the data is clearly described and appropriate, and the measurement of the association is presented, including confidence intervals and the probability level (p value) * 3. The statistical test is not appropriate, not described or incomplete (e.g. results from all rating scales performed should be described)   This scale has been adapted from the Newcastle-Ottawa Quality Assessment Scale^2^ for cohort studies to perform a quality assessment of cross-sectional studies for the systematic review. We have not selected one factor that is the most important for comparability, because the variables are not the same in each study. Thus, the principal factor should be identified for each study. The resulting quality score can be used to compare risk of bias across cross-sectional studies in our review.  Literature  ^1^Kloosterboer, S. M., Vierhout, D., Stojanova, J., Egberts, K. M., Gerlach, M., Dieleman, G. C., . . . Koch, B. C. P. (2020). Psychotropic drug concentrations and clinical outcomes in children and adolescents: a systematic review. *Expert Opin Drug Saf, 19*(7), 873-890. doi:10.1080/14740338.2020.1770224  ^2^Wells G, Shea B, O'Connell D, Peterson J, Welch V, Losos M, et al. The Newcastle-Ottawa Scale (NOS) for assessing the quality of nonrandomised studies in meta-analyses. Available from:  http://www.ohri.ca/programs/clinical_epidemiology/oxford.asp. |

Abbreviations

| Arbeitsgemeinschaft für Neuropsychopharmakologie und Pharmakopsychiatrie | AGNP |
| --- | --- |
| Clinical Global Impression Scale | CGI |
| Cytochrome P450 | CYP |
| Hamilton Depression Scale | HAMD |
| High performance liquid chromatography | HPLC |
| Liquid chromatography–mass spectrometry | LC-MS |
| Montgomery–Åsberg Depression Rating Scale | MADRS |
| Positron emission tomography | PET |
| Preferred Reporting Items for Systematic Reviews and Meta-Analyses | PRISMA |
| Preferred Reporting Items for Systematic Reviews and Meta-Analyses Protocols | PRISMA-P |
| Risk of Bias | RoB |
| Standard deviation | SD |
| Single photon emission computed tomography | SPECT |
| Target engagement | TE |
| Therapeutic drug monitoring | TDM |
| World Federation of Societies of Biological Psychiatry | WFSPB |
